# Transcriptome analysis reveals the effect of grafting on gossypol biosynthesis and gland formation in cotton

**DOI:** 10.1186/s12870-022-04010-z

**Published:** 2023-01-16

**Authors:** Kun Ye, Teng Teng, Teng Yang, Degang Zhao, Yichen Zhao

**Affiliations:** 1 College of Tea Sciences, College of Life Sciences, The Key Laboratory of Plant Resources Conservation and Germplasm Innovation in Mountainous Region (Ministry of Education), Guizhou University, Guiyang, 550025 China; 2 Plant Conservation Technology Center, Guizhou Key Laboratory of Agricultural Biotechnology, Guizhou Academy of Agricultural Sciences, Guiyang, 550006 China

**Keywords:** Cotton, Grafting, Pigment gland density, Gossypol content, Gossypol synthesis related genes, Transcriptome analysis, Differentially expressed genes, Real-time PCR analysis

## Abstract

**Background:**

Gossypol is a unique secondary metabolite and sesquiterpene in cotton, which is mainly synthesized in the root system of cotton and exhibits many biological activities. Previous research found that grafting affected the density of pigment glands and the gossypol content in cotton.

**Results:**

This study performed a transcriptome analysis on cotton rootstocks and scions of four grafting methods. The gene expression of mutual grafting and self-grafting was compared to explore the potential genes involved in gossypol biosynthesis. A total of six differentially expressed enzymes were found in the main pathway of gossypol synthesis-sesquiterpene and triterpene biosynthesis (map00909): lupeol synthase (LUP1, EC:5.4.99.41), beta-amyrin synthase (LUP2, EC:5.4.99.39), squalene monooxygenase (SQLE, EC:1.14.14.17), squalene synthase (FDFT1, EC:2.5.1.21), (-)-germacrene D synthase (GERD, EC:4.2.3.75), ( +)-delta-cadinene synthase (CADS, EC:4.2.3.13). By comparing the results of the gossypol content and the density of the pigment gland, we speculated that these six enzymes might affect the biosynthesis of gossypol. It was verified by qRT-PCR analysis that grafting could influence gene expression of scion and stock. After suppressing the expression of the *LUP1*, *FDFT1*, and *CAD* genes by VIGS technology, the gossypol content in plants was significantly down-regulated.

**Conclusions:**

These results indicate the potential molecular mechanism of gossypol synthesis during the grafting process and provide a theoretical foundation for further research on gossypol biosynthesis.

**Supplementary Information:**

The online version contains supplementary material available at 10.1186/s12870-022-04010-z.

## Background

Cotton (*Gossypium* spp.) is an annual herb native to subtropical regions. It is one of the most important crops worldwide, with the main characteristics of low production costs and high yield. Gossypol is a specific plant defense compound that cotton plants produce. It is a dimeric sesquiterpene, a secondary metabolite, and synthesized in the cytoplasm. The exploration of the gossypol biosynthetic pathway began decades ago, and many key enzymes and transcription factors were found to regulate the biosynthesis of gossypol. Chen et al. reported the first sesquiterpene cyclase cadinene synthase and then the first cytochrome P450 mono-oxygenase by Luo et al. [1, 2]. In the cytoplasmic matrix, farnesyl diphosphate (FPP) is synthesized from the mevalonate (MVA) pathway. Isopentenyl diphosphate (IPP) and dimethylallyl diphosphate (DMAPP) are catalyzed by farnesyl diphosphate synthase (FPS) to form FPP; then gossypol synthesis occurs under the catalytic action of several key enzymes such as ( +)-δ-cadinene synthase (CDN), transcription factor (GaWRKY1) and cytochrome P450 mono-oxygenase (CYP706B1) [3, 4].

Gossypol is mainly synthesized in cotton roots, transported to aboveground parts, and eventually stored in the pigment glands [5]. Pigment glands are a unique morphological feature of cotton (*Gossypium* spp.), which are distributed in most organs of cotton plants. Studies have shown that the gossypol synthesis pathways and the development of pigment glands are independent of each other. The inhibition of the development of pigment glands may also inhibit the biosynthesis of gossypol [6]. Gossypol biosynthesis is not directly related to pigment gland expression, but the presence of pigment glands is essential for gossypol accumulation [7].

Gossypol has a variety of functions in different sectors. In agriculture, it can inhibit the growth of cotton bollworm and other pests. It also has a certain inhibitory effect on the development of spores of a fungal species *Verticillium dahliae*, and an antifertility effect on rodents. In industry, depending on its chemical structure, gossypol can be used as an antioxidant, flame retardant, coloring agent, and stabilizer [8–11]. In addition, many studies have found that gossypol has antifertility, antioxidant, antitumor, antiviral, and other medicine activities [12–15].

Grafting is an agricultural technique used for the vegetative propagation of crops, as early as 2000 years ago in China, grafting has been used in horticulture [16]. The method involves grafting branches or buds (scions) of one plant onto the stems or roots (rootstocks) of another plant so that the parts of two different plants can grow into a new plant. It is widely used in cultivation and for the improvement of crops, due to its low cost, simple operation, high value of use, and other advantages [17]; for some difficult-to-graft plants, such as walnuts, factors such as temperature, humidity, and treatment methods of grafting materials must be taken into account when grafting [18]. Previous studies have found that the grafting technique is an effective way to study plant molecular communication signals and the exchange of genetic material [19]. The accumulation of biomass in the scion part and the response to abiotic stress can be changed depending on different rootstocks [20–22]. Li et al. (2021) found that using high disease-resistant varieties as rootstocks can up-regulate the expression of disease-resistant genes in scions, thereby improving the resistance of susceptible varieties [23], Zhang et al. (2019) used Artemisia-chrysanthemum as a rootstock to improve chrysanthemum resistance to aphids [24]. This study found that root exchange could affect gland density and gossypol content. The low-gossypol cotton root system caused a significant decrease in the gossypol content in the high-gossypol scion. When low-gossypol cotton was used as a scion, the gossypol content in the high-gossypol cotton rootstock increased significantly. We speculated that the root system plays an important role in gossypol synthesis. Therefore, we performed transcriptome sequencing to screen and analyze related genes involved in gossypol synthesis at the molecular level to provide a basis for further research on gossypol biosynthesis in cotton.

## Materials and methods

### Plant materials

**Fig. 1:**
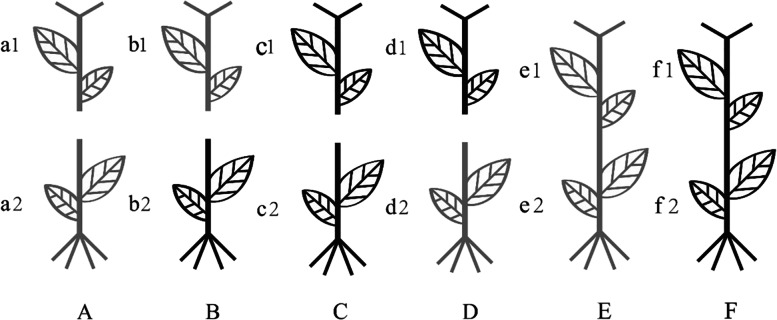
Four grafting methods. Gray color represents low-gossypol cotton, black color represents high-gossypol cotton; 1 Represents scion, and 2 Represents rootstock. **A** Scion and rootstock both are low-gossypol cotton **B**. Scion is low-gossypol cotton and rootstock is high-gossypol cotton **C**. Scion and rootstock both are high-gossypol cotton **D**. Scion is high-gossypol cotton and rootstock is low-gossypol cotton E. Ungrafted low-gossypol cotton F. Ungrafted high-gossypol cotton

The seeds of high-gossypol upland cotton (*Gossypium hirsutum* L. cv. S9612) and glandless low-gossypol upland cotton (*Gossypium hirsutum* L. cv. Zhong –151) used in this study were provided by the Cotton Research Institute of the Chinese Academy of Agricultural Sciences (Anyang, China). The cotton plants were grafted by splitting at the 4–5 leaves stage. Four months after grafting, samples were taken for related experiments. In this study, scion leaves were taken from the second unfolded leaf of each plant (counting was done from top to bottom); rootstock leaves were taken from the second true leaf of each plant. In total, four treatments were given in this study. The root systems of high-gossypol and low-gossypol cotton plants exchanged for grafting are shown in Fig. 1.

### The observation of pigment gland density

Four leaves and stems at the same growth stage were taken. Leaf samples were taken from different positions, including the right nectary, the left nectary, the middle vein, the middle left vein, the and right vein. The stem sample was dissected, and the internal tissue and the epidermis were removed. A stereo microscope was used to observe the samples. Five fields of view were randomly selected, the average value of these fields was considered as the stem gland density, and the data were statistically analyzed using Excel. Use "one-way ANOVA" to calculate the significance of the sample, In the output results, *p*-value < 0.05 indicates a significant difference, and *p*-value < 0.01 indicates a very significant difference.

### Determination of gossypol content by UPLC

The cotton samples were dried at 45 °C to a constant weight, broken up into small pieces, passed through a 60-mesh sieve, weighed (0.1 g; accurate to 0.0001 g), dissolved in acetonitrile-0.2% phosphoric acid aqueous solution, extracted by ultrasonic vibration for 15 min, and centrifuged at 1000 × g for 5 min. The supernatant was filtered through a 0.22 µm water-based membrane filter and stored at –20 °C in the dark. The UItiMate 3000 ultra-high performance liquid chromatography (Thermo, USA), with DAD-3000RS diode array detector (Thermo, USA), TCC-3000RS column thermostat (Thermo, USA), WPS-3000TRS autosampler (Thermo, USA), LPG-3400RS quaternary pump (Thermo, USA), and SR-3000 reagent rack (Thermo, USA), was used for the analysis. Separation was performed using a Hypersil GOLDTM C18 column (100 mm × 2.1 mm, 1.9 µm). The Mobile phase consisted of acetonitrile and 0.2% phosphoric acid aqueous solution (v/v). The mobile phase ratio was 90: 10, with a flow rate of 0.4 mL/min and a column temperature of 25 °C. The detection wavelength was 235 nm, and the injection volume was 10 µL. The gossypol standard product (purity ≥ 95%)was purchased from Sigma. The gossypol content was calculated based on the standard curve and the peak area of three biological replicates, and the data were analyzed using SPSS software.

### RNA extraction, library construction, and sequencing

Total RNA was extracted from leaves using Trizol reagent (TaKaRa, China) according to the manufacturer’s protocol. The purity and concentration of RNA were measured using the Ultra-micro full-wavelength reader (IMPLEN, Germany). The integrity of the RNA was determined by 2% agarose gel electrophoresis. The library construction and the RNA-Seq analysis were performed using the Illumina HiSeq X-ten platform by BGI.

### Filtering on sequencing reads and aligning with reference genome

Original data were filtered in three steps: removing reads containing connectors (adapter contamination), eliminating reads with poly-N proportions greater than 5%, and ignoring low-quality reads (the bases defined with quality values less than 10, accounting for more than 20% of the total bases of reads, were considered as low-quality reads) [25]. After getting the clean reads, HISAT was used to map the clean reads to the reference genome (reference genome version: GCF_000987745.1_ASM98774v1) [26].

### Functional annotation and classification

To annotate the unigenes, the BLAST software was used to search the unigenes against a large number of protein and nucleotide databases, including Kyoto Encyclopedia of Genes and Genomes (KEGG), Gene Ontology (GO), protein families (Pfam), eggNOG, and Clusters of Orthologous Groups (COG) [27–34].

### Analysis of DEG profiles

Unigene expression was normalized with FPKM (fragment per kilobase per million mapped reads), and then differential expression analysis of two sample groups was performed by DESeq [35]. The ratios of FPKM values of different samples were calculated for DGEs. FDR (false discovery rate) was used to determine the threshold of the *p*-value for multiple tests. FDR ≤ 0.001 and the absolute value of │log2 ratio│ ≥ 1 were considered to be the cutoff thresholds to determine the significance of expression. The DEGs involved in gossypol synthesis were screened, and GO and KEGG databases were used to annotate and classify DEGs.

### Quantitative Real-Time PCR

QRT-PCR was used to verify the expression of the selected DEGs, and GhUbiquitin (ghUBQ14, GenBank accession number: DW505546) was used as an internal reference gene. We used Quick RNA isolation Kit (Waryong, Beijing, China) to extract RNA from eight grafted cotton samples and detected the purity and concentration of RNA by agarose gel electrophoresis. If the RNA quality was good, PrimeScript RT reagent Kit (TaKaRa) was used for reverse transcription into cDNA. A 10-µL aliquot of qRT-PCR mixture contained: SYBR qPCR SuperMix Plus 5 µL, template cDNA 1 µL, forward and reverse primers 0.3 µL each, and ddH_2_O 3.4 µL, the primer sequences were listed in Table 1. The delta-delta CT method was used to compare the relative expression levels of genes.

**Table 1:** Primer for qRT-PCR

Gene	Forward primer sequence(5’-3’)	Forward primer sequence(3’-5’)
*UBQ14*	CAACGCTCCATCTTGTCCTT	TGATCGTCTTTCCCGTAAGC
*LUP1*	AGATGGTGAGGAAATGGCTG	GGAAAGAGGTTAGGTAGAAGC
*LUP2*	CGGTGACGGTGAGATTAGATAC	AGGTGAATTGTTAAGGGTGGG
*GERD*	CTAGGGCTCCATTTCCAGTTC	AACAGTGAAGTAATCCCAGCC
*FDFT1*	AGGAAAACTCGGTCAAGGC	ACACCTCGGAAGACTTTGATG
*SQLE*	GGGTATTTGAGACTTGGAGGG	ATTTAGCCCCATCCCACAAG
*CAD*	ACTGTGGGATTGCCTAATAAGCC	TTGCCACATCAATCACAAATCG

### Functional characterization of the candidate genes through virus-induced gene silencing

The online tool SGN VIGS (https://vigs.solgenomics.net/) was used to design the appropriate silencing region in the target gene [36]. The parameters were set as follows: n-mer size is 21, fragment length is 300, and mismatches is 0. The specific parameters could be adjusted according to the score. DNAMAN8 was used for restriction enzyme analysis, the target fragment was ligated to TRV2 (tobacco rattle virus) vector through EcoRI and BamHI digestion sites, and the recombinant vector was constructed into the competent cells of *Agrobacterium tumefaciens* GV3101 by freeze–thaw method. The bacterium GV3101 containing pTRV1 vector was used as auxiliary bacteria, TRV1/GV3101 and TRV2/GV3101 were mixed in a 1:1 ratio and injected into the cotyledons of 8-day-old cotton seedlings until the leaves were filled and incubated in the dark for 24 h, at least 30 cotton seedlings were injected for each gene. After 30 days, the leaves were harvested to measure the gossypol content.

## Results

### The observation of pigment gland density

**Fig. 2:**
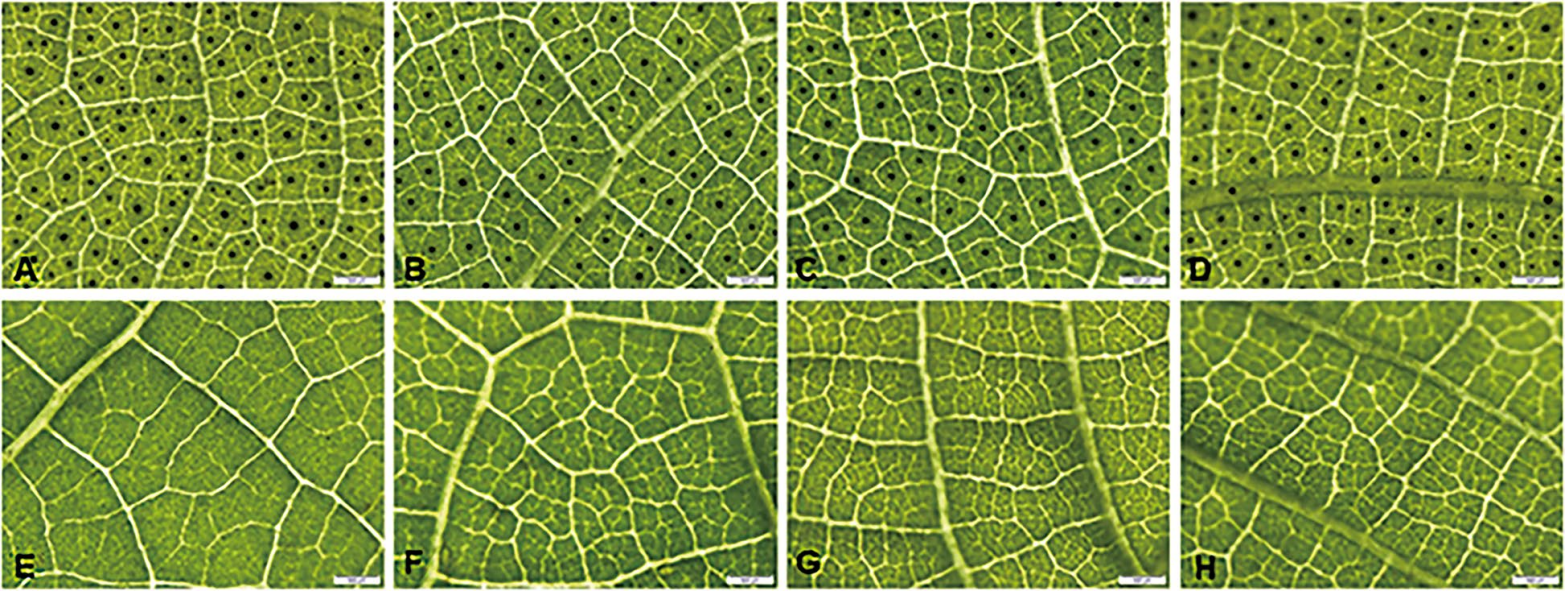
The formation of pigment glands in leaves of low-gossypol cotton and high-gossypol cotton after root exchange (bar = 500 µm); **A** leaves of b2, **B** leaves of c1, **C** leaves of c2, **D** leaves of d1, **E** leaves of a1, **F** leaves of a2, **G** leaves of b1, and **H** leaves of d2

The observation of the gland density of eight grafted leaves in four groups showed that the glands appeared in high-gossypol cotton leaves in different treatments (Fig. 2A-D) but were not formed in low-gossypol cotton leaves in all treatments (Fig. 2E-H), indicating that the roots of low-gossypol and high-gossypol cotton plants were exchanged for four months. The phenotype of the low-gossypol cotton with no glands was not significantly affected by the roots of high-gossypol cotton plants.

**Fig. 3:**
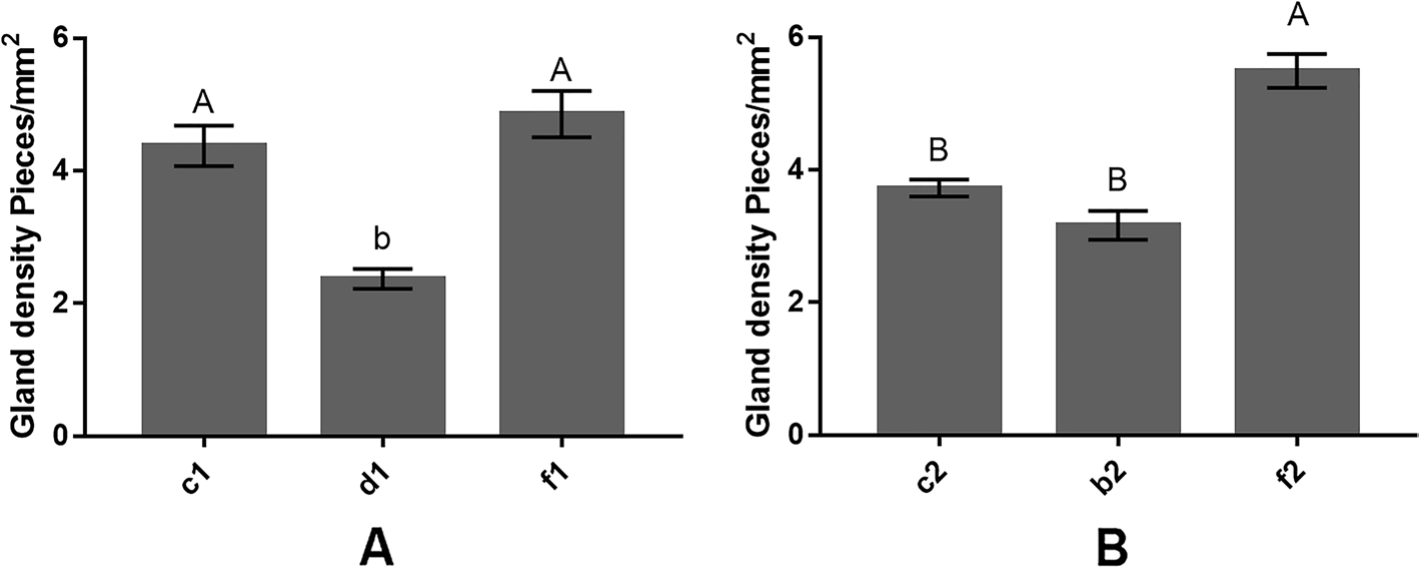
The gland density in leaves of the high-gossypol cotton plants; **A** The gland density in different rootstocks and scion of the high-gossypol cotton, f1 is the scion of the ungrafted high-gossypol cotton. **B** The gland density in different rootstocks and scion of the high-gossypol cotton, f2 is the rootstock of the ungrafted high-gossypol cotton. Different letters indicate differences at *P* < 0.05, while uppercase letters indicate differences at *P* < 0.01

In the rootstock, the gland densities of the self-grafting, mutual grafting and ungrafting high gossypol cotton leaves were: 53.83 ± 3.71, 45.95 ± 3.16 and 79.78 ± 5.49, respectively. In the scion, the gland densities of the self-grafting, mutual grafting and ungrafting high gossypol cotton leaves were: 34.43 ± 2.37, 63.58 ± 4.38 and 70.50 ± 4.86, respectively. Statistical analysis for the density of glands in the leaves of high-gossypol cotton showed that the density of pigment glands in leaves of ungrafted high-gossypol cotton was significantly higher than that of high-gossypol cotton with both their individual roots and exchanged roots, indicating that grafting can reduce the density of pigment glands in leaves. After the exchange of roots from high-gossypol and low-gossypol cotton plants, the low-gossypol cotton root system caused a significant decrease in the density of the pigment glands in the leaves of the high-gossypol cotton scion, but when low-gossypol cotton was used as the scion, the changes in pigment glands in the rootstock leaves of high-gossypol cotton were not significant (Fig. 3). This indicates that the root system of low-gossypol cotton can cause a significant decrease in the density of pigment glands in high-gossypol cotton, confirming that the density of pigment glands can be generally affected by the root system.

### Determination of gossypol content by UPLC

**Fig. 4:**
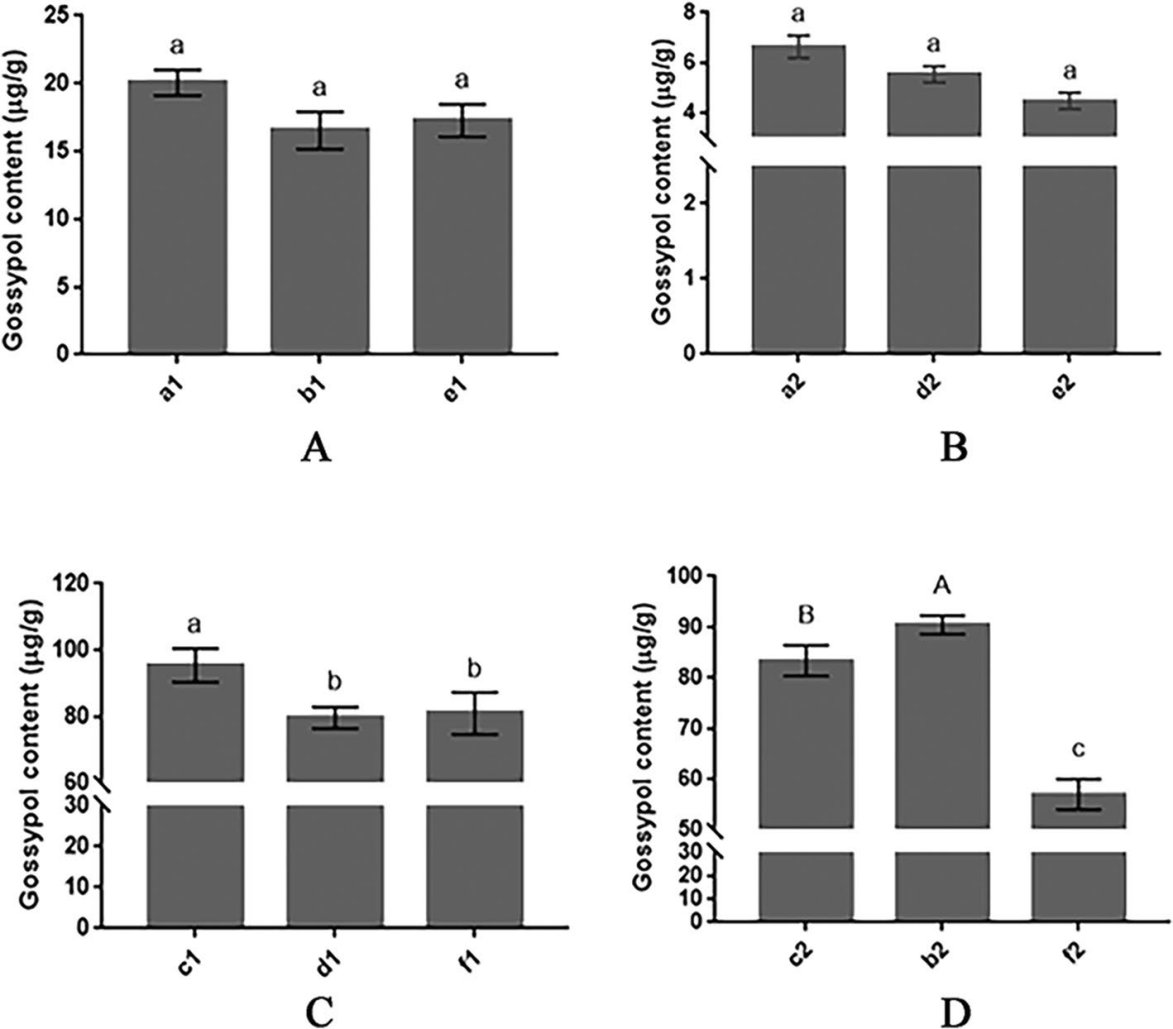
**A** The gossypol content in the scion of different rootstocks of low- gossypol cotton; e1 is the scion of the ungrafted low-gossypol cotton, **B** The gossypol content in the rootstock of different scions of low-gossypol cotton, e2 is the rootstock of the ungrafted low-gossypol cotton. **C** The gossypol content in the scion of different rootstocks of high-gossypol cotton, f1 is the scion of the ungrafted high-gossypol cotton. **D** The gossypol content in the rootstock of different scions of high-gossypol cotton, f2 is the rootstock of the ungrafted high-gossypol cotton. Different letters indicate differences at *P* < 0.05, while uppercase letters indicate differences at *P* < 0.01

UPLC was used to determine the gossypol content in eight samples of four grafted scions and rootstocks groups. The results of the analysis are shown in Fig. 4. The results of the analysis of the gossypol content in the leaves of low-gossypol cotton plants grafted onto different roots revealed that the gossypol content in the leaves of low-gossypol cotton was much lower than that of high-gossypol cotton. The gossypol content in low-gossypol cotton did not change significantly with both their individual roots and interchanged roots (Fig. 4A, B). This indicates that the root system of low-gossypol cotton has a weak ability to synthesize gossypol and lacks pigment glands. The presence or absence of pigment glands can seriously affect the gossypol content.

The results of the determination of the gossypol content in the leaves of high-gossypol cotton grafted onto different root systems revealed that the gossypol content of the leaves of ungrafted high-gossypol cotton was lower than that of the scion leaves of high-gossypol cotton with their individual roots and interchanged roots, indicating that grafting could increase the gossypol content (Fig. 4C, D). After exchanging the roots of high-gossypol and low-gossypol cotton plants, the root system of low-gossypol cotton caused a significant decrease in the gossypol content in the scion leaves of high-gossypol cotton (Fig. 4C). When low-gossypol cotton was used as the scion, the gossypol content in the rootstock leaves of high-gossypol cotton increased significantly (Fig. 4D). This observation indicated that the root system of the low-gossypol cotton has a weaker ability to synthesize gossypol than that of the high-gossypol cotton, and the low-gossypol cotton cannot store gossypol due to the lack of pigment glands, increasing the gossypol content in the rootstocks of the high-gossypol cotton.

### Illumina Sequencing and data filtering

By sequencing using the Illumina HiSeq × platform, a total of 22,922,599, 22,748,498, 22,810,132, 22,885,527, 22,850,618, 22,858,512, 22,627,110, and 22,701,936 clean reads were obtained from eight samples of four grafted cotton groups (Table 2). The values of Q20 and Q30 were higher (95.67–99.13%), indicating that the results of the RNA-seq analysis met the requirements for the quality evaluation for subsequent analyses. After obtaining the clean reads, the HISAT was used to align the clean reads to the reference genome sequence. The difference between the efficiency of clean reads and the reference genome of eight samples was greater than 90%, indicating that the high utilization of gene expression profiling data and other data was sufficient for subsequent analysis.

**Table 2:** The summary of the mapping of the transcriptome reads onto the reference sequence in grafted cotton plants

Sample	Total Clean Reads(M)	Clean Reads Radio(%)	Clean Reads Q20 (%)	Clean Reads Q30 (%)	Total Mapped	Total Mapping(%)
a1	22,922,599	96.85	99.07	96.59	19,917,446	94.98
a2	22,748,498	95.65	98.98	96.23	19,659,252	94.83
b1	22,810,132	96.40	99.13	96.59	19,733,045	94.65
b2	22,885,527	96.70	99.12	96.45	19,800,557	94.86
c1	22,627,110	95.14	99.01	96.2	19,674,272	94.57
c2	22,701,936	95.46	98.98	96.2	19,621,283	94.13
d1	22,850,618	96.09	98.95	95.67	19,964,585	94.92
d2	22,858,512	96.60	99.12	96.67	20,014,913	94.82

### Functional annotation and categorization

BLAST software was used to annotate all unique sequences in nucleotide and protein databases to obtain complete functional annotations, including KEGG, GO, Pfam, eggNOG, and COG. Among these unigenes, 71,896 unigenes were annotated in at least one database. The unannotated unigenes may be specific to cotton or the homologous counterparts in other species, and their biological functions have not yet been studied. The number of genes expressed in each sample and the success rate of gene annotation in each database are shown in Table 3.

**Table 3:** The summary of functional annotation of unigenes in Gossypium hirsutum Linn

Database	Total unigene	Annotated unigenes	Percent
KEGG Pathway	71,896	27,600	38.39%
KEGG Disease	71,896	11,553	16.07%
KEGG Module	71,896	11,394	15.85%
KEGG Reaction	71,896	11,011	15.32%
GO C	71,896	22,876	31.82%
GO F	71,896	34,129	47.47%
GO P	71,896	27,848	38.73%
Pfam	71,896	50,599	70.38%
EggNOG	71,896	35,860	49.88%
COG	71,896	6713	9.34%

GO is an international classification system for standardized gene functions. The unigenes were assigned to GO terms for the functional classification. A total of 71,896 unigenes were divided into three categories: biological process, cellular component, and molecular function. The largest category was the cellular component, with 64,682 unigenes, followed by the biological process (64,569 unigenes) and the molecular component (46,641 unigenes). The metabolic process and binding were the two largest biological process and molecular function categories subcategories, respectively (Fig. S1). The results of the GO analysis showed that the unigenes identified by sequencing were involved in a series of biological processes.

To identify active biological pathways during the grafting process, a total of 47 pathways were predicted by searching for unigene sequences against the collection of pathways in the KEGG database. The most representative pathways include signal transduction, global and overview maps, immune system, and 1371 unigenes were related to the biosynthesis of other secondary metabolites (Fig. S2).

### Differential gene expression analysis

**Fig. 5:**
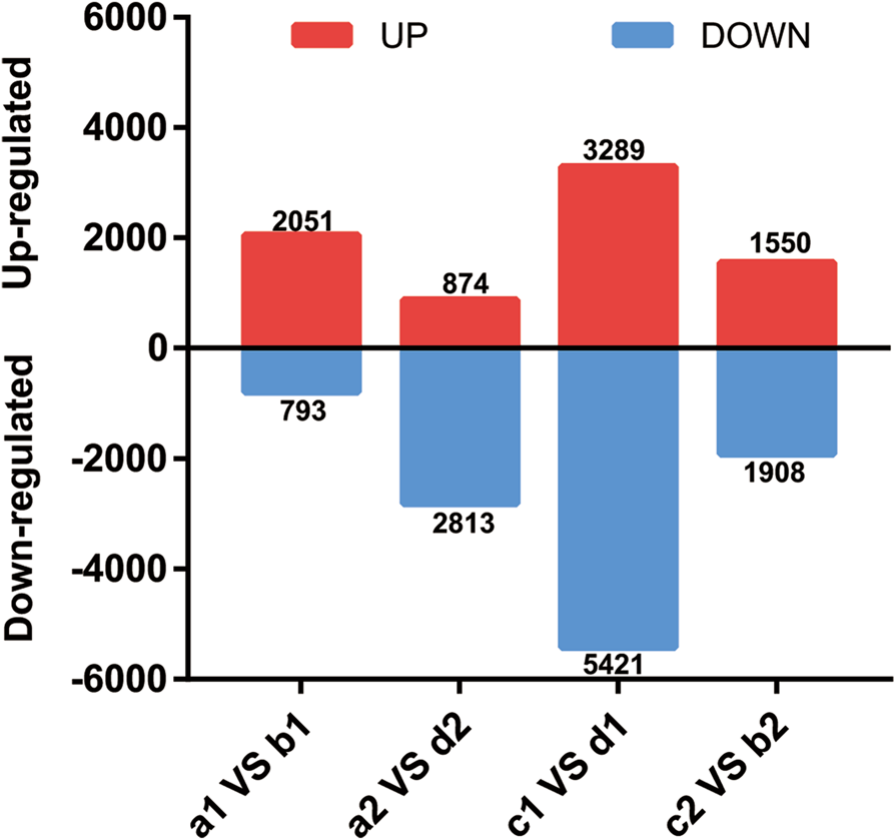
The bar graph of up- and down-regulated genes from pairwise comparison

To further examine the effect of grafting on the content of gossypol, we analyzed the transcriptomes of grafting of eight samples onto four plant groups. Comparative transcriptome analysis of the same part and various cotton plants was performed. A total of 2844 DEGs, including 2051 positively regulated and 793 negatively regulated genes, were detected from a1vsb1. Furthermore, 3689 DEGs, including 874 positively regulated and 2813 negatively regulated genes, were detected from a2vsd2 and 8710 DEGs, including 3289 positively regulated and 5421 negatively regulated genes, were detected from c1vsd1. Finally, 3458 DEGs, including 1550 positively regulated and 1908 negatively regulated genes, were detected from c2vsb2 (Fig. 5). When low-gossypol cotton was grafted on high-gossypol cotton rootstock, the number of up-regulated genes was much higher than those of down-regulated genes. Similarly, when high-gossypol cotton was grafted on low-gossypol cotton rootstock, the number of down-regulated genes was much more than that of up-regulated genes.

**Fig. 6:**
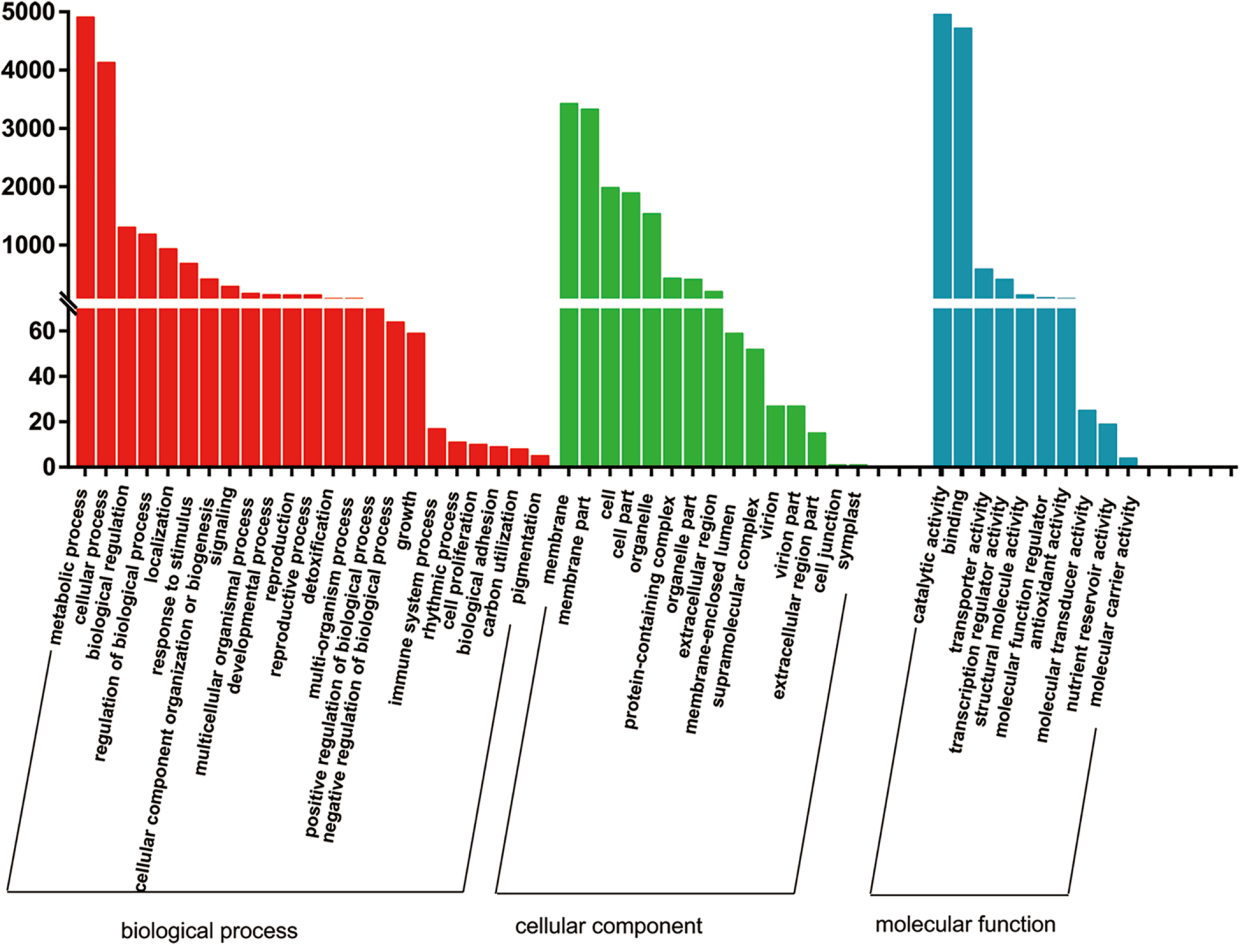
The GO classification of DEGs. In this figure, the abscissa represents the secondary classification of GO terms; the ordinate represents the number of genes associated with the secondary classification; and the three colors represent three classifications, including biological processes (red), cellular component (green), and molecular function (blue)

GO enrichment analysis of DEGs was performed to reveal DEGs and the related pathways under different grafting treatments. A total of 9817 DGEs were classified into 48 functional groups, including biological process (23), cellular component (15), and molecular function (10). The largest category was the biological process (14,878 DGEs), followed by the cellular component (13,402 DGEs), and molecular function (11,045 DGEs). The main GO terms were catalytic activity, metabolic process, and binding (Fig. 6).

**Fig. 7:**
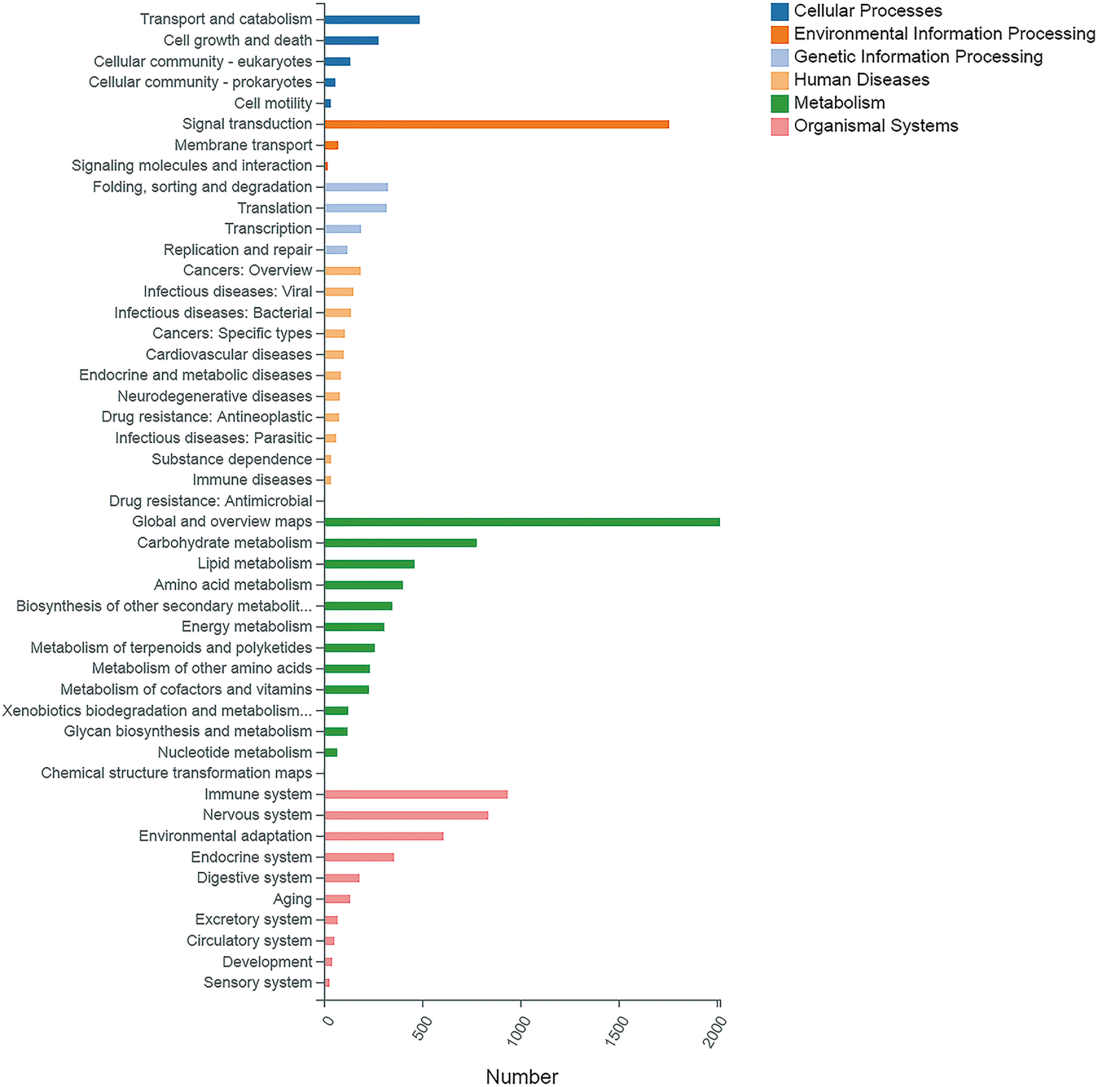
The KEGG annotation of DEGs. Permissions to use the KEGG pathway map was taken from the Kanehisa Laboratories (https://www.kanehisa.jp/)

The KEGG database was used to annotate the function of DEGs. A total of 27,600 DEGs were annotated into 47 pathways, including cellular processes, environmental information processing, genetic information processing, human diseases, metabolism, and organismal systems. Metabolism with 13 pathways was the largest category, and 348 DEGs were related to the biosynthesis of other secondary metabolites. The number of DEGs participating in categories such as global and overview maps, signal transduction, and carbohydrate metabolism was the largest. This indicates that more DEGs were involved in these pathways that were enriched in grafted cotton, providing useful information on the regulatory mechanism of grafted cotton (Fig. 7).

### The selection of putative genes related to gossypol synthesis

Gossypol is a unique secondary metabolite and a sesquiterpene in cotton. Through the KEGG annotation of DEGs of the same part of the same cotton varieties after grafting, we found that a large number of DEGs participated in sesquiterpenoid and triterpenoid biosynthesis (map00909). Sesquiterpenoids (C15 terpenoids) are a group of terpenoids consisting of three isoprene units. They are derived from FPP and can undergo cyclization to produce various skeletal structures. Sesquiterpenoid biosynthesis begins with the loss of diphosphate from FPP under the action of sesquiterpene synthesis enzymes, generating an allylic cation that is highly susceptible to intramolecular attacks. The cyclization of the farnesyl cation may take place on either of the remaining double bonds, resulting in the formation of 6-, 10-, or 11-membered rings [37–39].

**Fig. 8:**
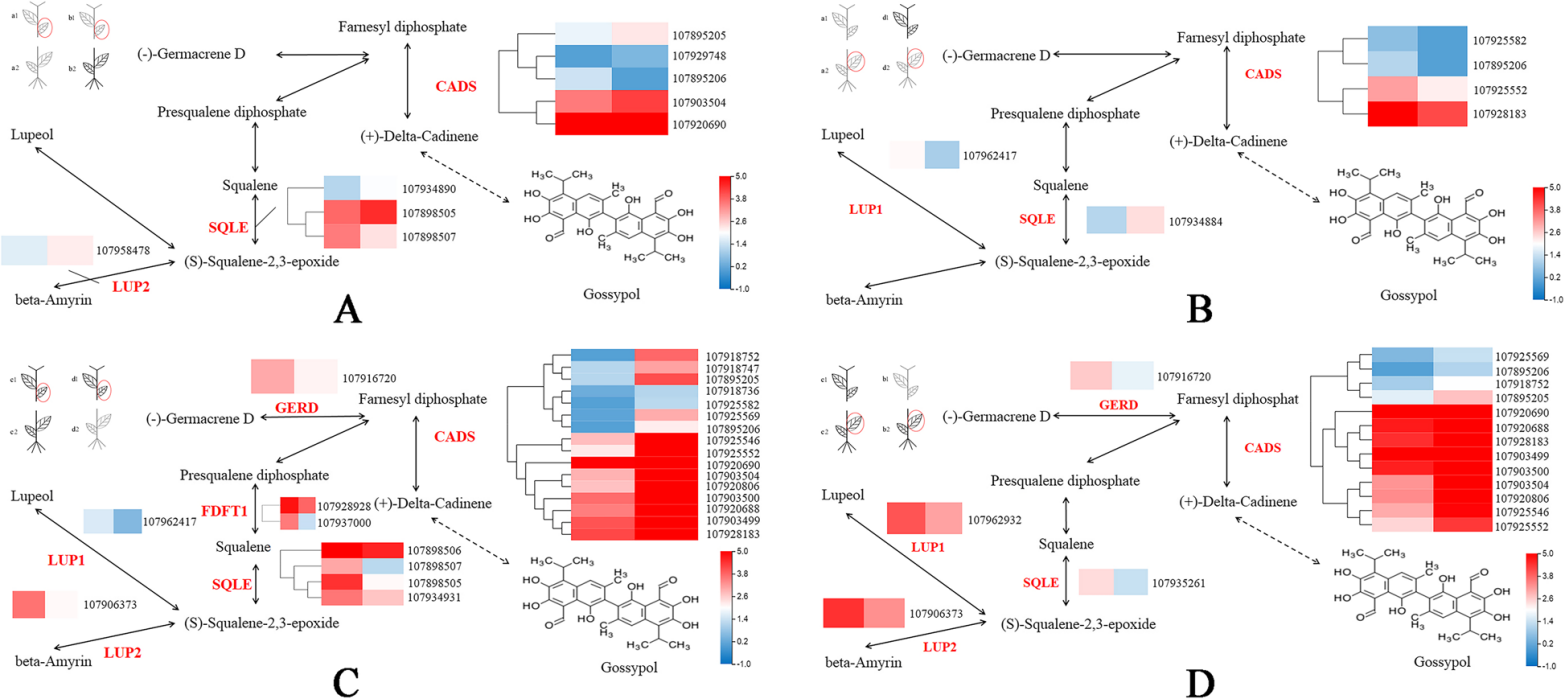
**A** The expression of six enzyme-related genes in a1 VS b1; **B** The expression of six enzyme-related genes in a2 VS d2; **C** The expression of six enzyme-related genes in c1 VS d1; **D** The expression of six enzyme-related genes in c2 VS b2

In this study, we found that a total of six enzymes, including lupeol synthase (LUP1, EC:5.4.99.41), beta-amyrin synthase (LUP2, EC:5.4.99.39), squalene monooxygenase (SQLE, EC:1.14.14.17), squalene synthase (FDFT1, EC:2.5.1.21), (-)-germacrene D synthase (GERD, EC:4.2.3.75), and ( +)-delta-cadinene synthase (CADS, EC:4.2.3.13), were differentially expressed in the pathways of sesquiterpenes and triterpenoid biosynthesis (map00909) (Fig. 8). The clustering heat map analysis of their expression levels revealed that most differentially expressed genes were located in the high-gossypol part of grafted plants. Among them, CADS is considered to be a key enzyme involved in the synthesis of gossypol [40, 41]. The screened differentially expressed CADS mainly belong to two subfamilies, including CDN1-A and CDN1-C. The expression level of most of these enzymes increased significantly in the high-gossypol part of the grafted plants, while it partly increased and then decreased in the low-gossypol part.

### Quantitative Real-Time PCR Analysis for the Selected DEGs

**Fig. 9:**
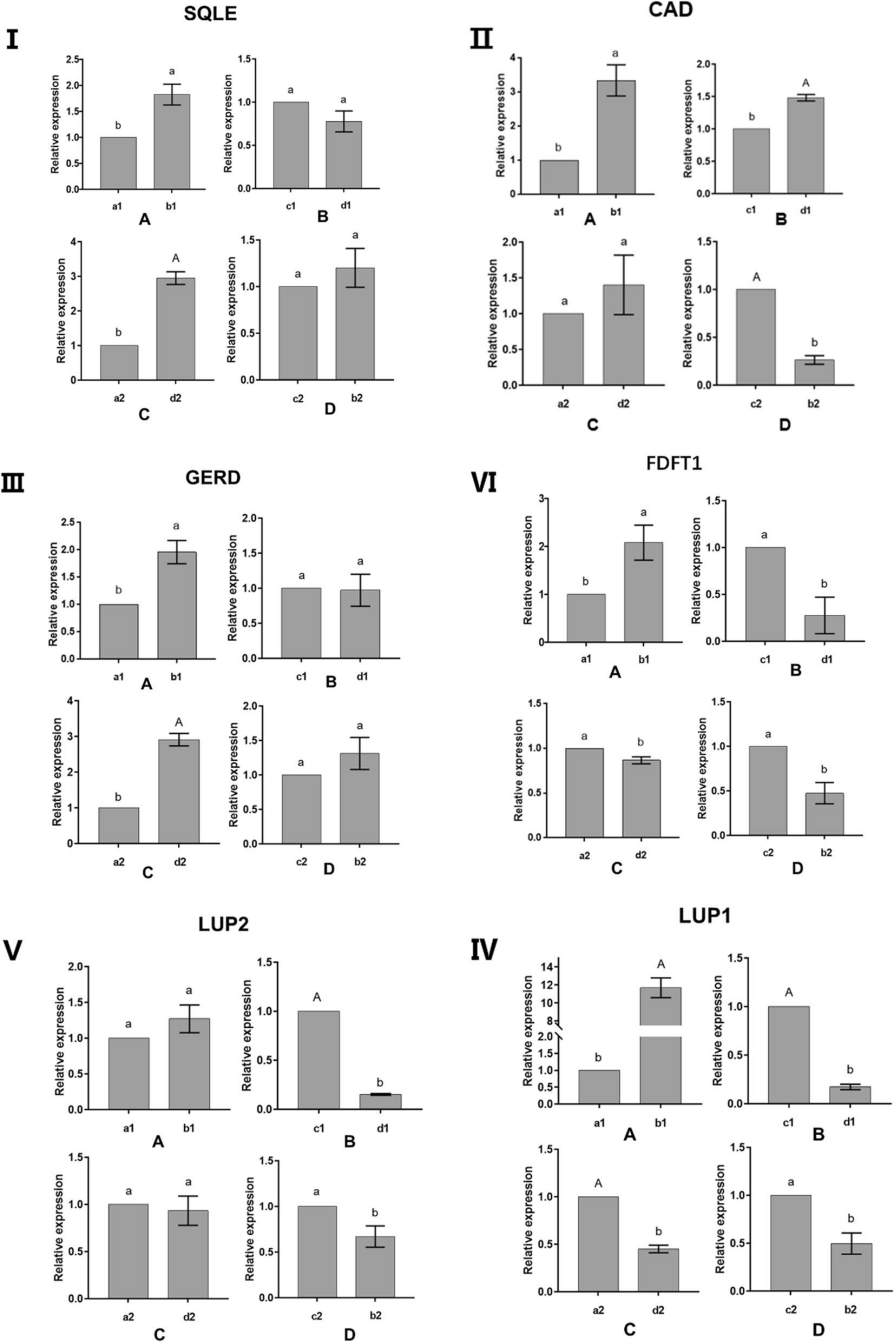
I. Relative expression of *SQLE* in rootstock or scion; II. Relative expression of *CAD* in rootstock or scion; III. Relative expression of *GERD* in rootstock or scion; IV. Relative expression of *LUP1* in rootstock or scion; V. Relative expression of *LUP2* in rootstock or scion; VI. Relative expression of *FDFT1* in rootstock or scion. **A** Low-gossypol scion grafted on low-gossypol rootstock or high-gossypol rootstock. **B** High-gossypol scion grafted on high-gossypol rootstock or low-gossypol rootstock. **C** Low-gossypol rootstock grafted with low-gossypol scion or high-gossypol scion. **D** High-gossypol rootstock grafted with high-gossypol scion or low-gossypol scion. **a** Low-gossypol cotton scion / low-gossypol cotton rootstock **b**. Low-gossypol cotton scion / high-gossypol cotton rootstock **c**. High-gossypol cotton scion / high-gossypol cotton rootstock **d**. High-gossypol cotton scion / low-gossypol cotton rootstock. 1. Scion 2. Rootstock

In order to further analyze the differences in the expression levels of genes related to gossypol synthesis, we used qRT-PCR to detect the expression of the six selected genes (*LUP1*, *LUP2*, *SQLE*, *FDFT1*, *GERD*, *CADS*) selected under four grafting methods (Fig. 9). As the figure shows, when low-gossypol cotton was connected with high-gossypol cotton, the expression of most genes in low-gossypol cotton increased significantly or very significantly. For example, when low-gossypol cotton was connected with high-gossypol cotton rootstock, the expression of the other five genes increased significantly except *LUP2*. When high-gossypol cotton was connected with low-gossypol cotton, the expression of most genes in high-gossypol cotton decreased significantly or very significantly. For example, when high-gossypol cotton was grafted with low-gossypol cotton, the expression of the other four genes decreased significantly except *SQLE* and *GERD*.

### Knockdown of *LUP1*, *FDFT1* and *CAD* genes in cotton through VIGS

**Fig. 10:**
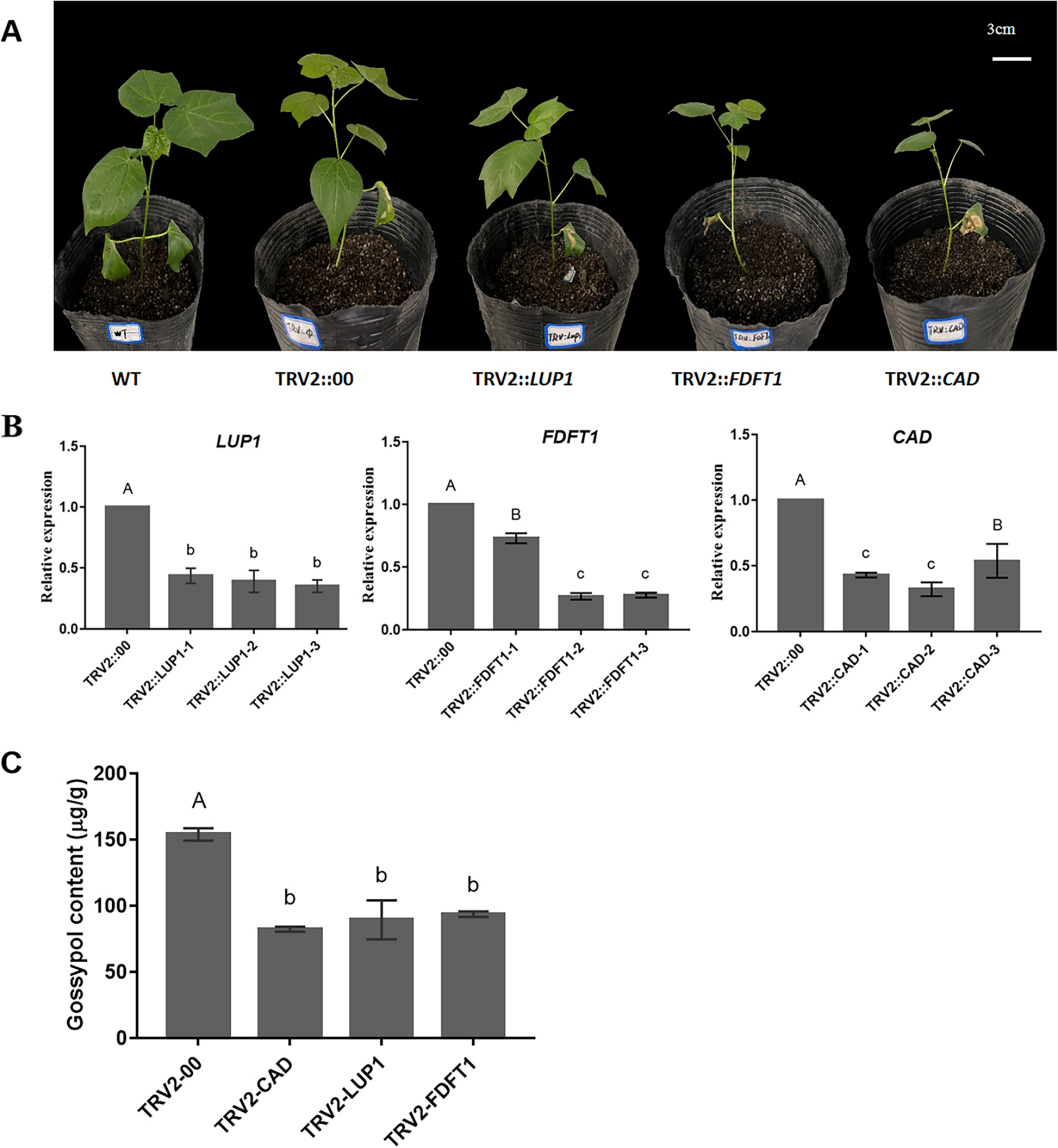
Virus-induced gene silencing (VIGS), **A** Morphological analysis of VIGS plants; **B** qRT-PCR was used to detect the expression of knockdown genes (*CAD, LUP1* and *FDFT1*) in VIGS plants; **C** Detection of Gossypol Content in VIGS Plants. In (**B**) and (**C**), each experiment was repeated three times. Error bars for gossypol content measurements represent standard deviations of three biological replicates, different letters indicate differences at *P* < 0.05, while uppercase letters indicate differences at *P* < 0.01

In order to further verify the role of these genes in the gossypol synthesis pathway, this study used HPLC to determine gossypol in the leaves of VIGS plants, and verified the actual function of these genes through their content changes, the results are shown in Fig. 10. Unfortunately, seven days after inoculation, albino leaves were not observed in TRV2:: *PDS* plants, which may be because the designed silencing site was not accurate enough to silence the *PDS* gene. Morphologically, 30 days after inoculation, the phenotypes of wild-type and empty plants were not much different. At the same time, TRV2::*CAD*, TRV2::*FDFT1* and TRV2::*LUP1* showed slow growth (Fig. 10A). The expression levels of *CAD*, *LUP1* and *FDFT1* in VIGS plants were detected by qRT-PCR. Their expression levels were down-regulated compared with the expression levels in leaves of TRV2::00 plants (Fig. 10B). Finally, the gossypol content in leaves was determined by HPLC. Compared with the TRV2::00 plants, the gossypol content in the leaves of the TRV2::*CAD*, TRV2::*LUP1* and TRV2::*FDFT1* plants decreased by 46.56%, 41.91% % and 39.12% respectively (Fig. 10C).

## Discussion

Transcriptome sequencing technology has been gaining popularity in functional genomics studies [42], which is an effective tool to help researchers identify transcriptome structure, differential gene expression, and alternative splicing, especially for cotton species with a complete reference genome [43]. Recently, many studies have been conducted on identifying genes related to fiber yield, flowering time, fiber length, and fiber quality in cotton using high-throughput sequencing technology [44, 45]. The transcriptome sequencing can generally be used to gain insight into the evolutionary genomics of cotton domestication [46]. This study used transcriptome sequencing to identify differentially expressed genes (DEGs) of cotton rootstocks and scions with different grafting methods. The DEGs were then annotated and classified, and the genes related to gossypol synthesis during the grafting process were screened. These results laid the foundation for studying the related molecular mechanisms of gossypol synthesis during the grafting process.

Gossypol is a unique secondary metabolite in cotton and is a sesquiterpene with several biological activities. Grafting is a commonly used method in agriculture, which can improve the ability of plants to withstand external diseases and insect pests [47]. According to recent research reports, grafting can be used as an effective way to study plant molecular communication signals and the exchange of genetic material, Liu et al. (2022) analyzed the metabolic mechanism of pumpkin rootstock mRNA signaling involved in regulating the cold tolerance of grafted cucumber [48]. Here, in the initial experiments, we measured the gland density and gossypol content after grafting. The results showed that although the root system of high gossypol cotton had good synthesis ability, the gossypol transported upward could not be stored due to the lack of glands in low gossypol cotton, so it was accumulated in the high gossypol cotton rootstock; at the same time, the gossypol synthesis ability of the low gossypol cotton root is weak, so it is unable to synthesize enough gossypol and transport it to the high gossypol cotton scion, which leads to the decrease of gossypol content and gland density in the scion. It seems that grafting has indeed affected the transportation of gossypol to a certain extent. Therefore, based on the results of the analysis of gland density and gossypol content, a transcriptome sequencing study on different parts with grafted plants was conducted, and the related genes involved in gossypol biosynthesis were further explored, screened, and regulated at the molecular level, thereby providing a theoretical basis for further research on the biosynthesis of gossypol.

A total of 8 transcriptome databases were constructed using the grafted samples. After sequencing using the Illumina HiSeq × platform, the raw reads were filtered. Each sample obtained about 22,800,000 clean reads, and Q20 and Q30 also met the analytical requirements and then used HISAT to compare the clean reads to the reference genome.

Through the screening of differentially expressed genes, it was found that most of the DEGs in high-gossypol cotton were down-regulated, and KEGG analysis showed that most of these genes were related to signal transduction and compound metabolism, which indicated that some plant hormone signal responses and changes in the metabolic pathways of compounds play an important role in the regulation of gossypol synthesis. Further functional annotation and classification of DEGs participating in sesquiterpenoid and triterpenoid biosynthesis (map 00,909) (part of the gossypol synthesis pathway), were performed, and six enzymes, including LUP1 (EC.5.4.99.41), LUP2 (EC.5.4.99.39), SQLE (EC.1.14.14.17), FDFT1 (EC.2.5.1.21), GERD (EC.4.2.3.75), and CADS (EC.4.2.3.13) involved in the synthesis of gossypol, were found. Cluster analysis of the expression level of related genes revealed different gene expression patterns, indicating that they may be gossypol synthesis-related genes.

From the results of real-time qRT-PCR, during the grafting process, it was found that the gene expression in the rootstock or scion after the grafting of high-gossypol cotton and low-gossypol cotton did have a significant difference compared with that of self-grafting. In general, the gene expression in the low-gossypol cotton is affected by the high-gossypol cotton and is significantly up-regulated. The gene expression in the high-gossypol cotton is affected by the low-gossypol cotton and is significantly down-regulated. The interaction between rootstock and scion also appeared in previous studies, such as *Arabidopsis thaliana* [49], potato [50], grape [51], etc., and this phenomenon may be caused by the mutual movement of some biological macromolecules between the two parts [52, 53]. Therefore, we speculate that high-gossypol cotton may transmit certain signaling molecules through the conduit, thereby stimulating the expression of gossypol synthesis-related genes in low-gossypol cotton. Similarly, there were signal molecules in low-gossypol cotton that can affect the gene expression of high-gossypol cotton. This confirms once again that grafting can be an ideal method for studying long-distance signal transmission in plants. However, there were some special cases, such as CAD, which is a key enzyme involved in the synthesis of gossypol. This study found that when the high-gossypol cotton scion was grafted on the low-gossypol cotton rootstock, the gene expression of the high-gossypol cotton scion was significantly increased. The gossypol content measurement results showed that the gossypol content of high-gossypol cotton scion was significantly decreased. In the subsequent VIGS experiments, VIGS vectors of these six genes were constructed, but plants died soon after *LUP2*, *SQLE*, and *GERD* were knocked down. This may be because these genes are not only involved in the synthesis of gossypol, but also plays an important role in growth and development. After the gene expression levels of *LUP1*, *FDFT1* and *CAD* were knocked down, the gossypol content in VIGS plants was significantly down-regulated compared with empty plants. This also proves again that these three genes play an important role in gossypol synthesis. On the basis of these results, it is speculated that the root system is the main place for gossypol synthesis. However, the low-gossypol cotton rootstock had a weak gossypol synthesis ability, resulting in a decrease in the gossypol content in the high-gossypol cotton scion and also stimulated the rootstock part of the gossypol content. The expression of gossypol synthesis gene led to a significant increase in its expression. These results reveal the potential molecular mechanism of gossypol synthesis during the grafting process and provide a theoretical basis for further research on the biosynthesis of gossypol and the potential connection between the rootstock and the scion during grafting.

## Conclusion

Our study demonstrated that the root system is the main source of gossypol synthesis, and pigment glands can also affect the accumulation of gossypol. Using transcriptome analysis, a total of six differentially expressed enzymes were found in the main pathway of gossypol synthesis-sesquiterpene and triterpene biosynthesis (map00909), which may affect the biosynthesis of gossypol. In addition, this study also found that after grafting, there may be some signal communication between the rootstock and the scion to regulate the expression of gossypol-related genes.

## Supplementary Information

**Additional file 1: Fig. S1.** GO classification of unigenes. In this figure, the abscissa represents the secondary classification of GO terms; the ordinate represents the number of genes associated with the secondary classification; and the three colors represent three classifications, including biological processes (red), cellular components (green), and molecular function (blue). **Fig. S2.** The KEGG annotation of unigenes. Permissions to use the KEGG pathway map was taken from the Kanehisa Laboratories (https://www.kanehisa.jp/).

## Data Availability

Data supporting the findings of this study are available from the corresponding author on a reasonable request.

## Abbreviations

KEGG: Kyoto Encyclopedia of Genes and Genomes
GO: Gene Ontology
LUP1: Lupeol synthase
LUP2: Beta-amyrin synthase
SQLE: Squalene monooxygenase
FDFT1: Squalene synthase
GERD: (-)-Germacrene D synthase
CADS: ( +)-Delta-cadinene synthase
HPLC: High-performance liquid chromatography
COG: Cluster of Orthologous Groups
GO: Gene Ontology
BP: Biological process
CC: Cellular component
MF: Molecular function
DEGs: Differentially expressed genes
qRT-PCR: Real-time Quantitative polymerase chain reaction
VIGS: Virus-induced gene silencing
