# Supplementary material for: Transcriptome analysis reveals the effect of grafting on gossypol biosynthesis and gland formation in cotton
Source: BMC Plant Biol. 2023 Jan 16;23:37. doi: 10.1186/s12870-022-04010-z (PMC9841644; doi:10.1186/s12870-022-04010-z)

Fig.S1. GO classification of unigenes. In this figure, the abscissa represents the secondary classification of GO terms; the ordinate represents the number of genes associated with the secondary classification; and the three colors represent three classifications, including biological processes (red), cellular components (green), and molecular function (blue).

Fig.S2. The KEGG annotation of unigenes. Permissions to use the KEGG pathway map was taken from the Kanehisa Laboratories (https://www.kanehisa.jp/).

Fig.S1


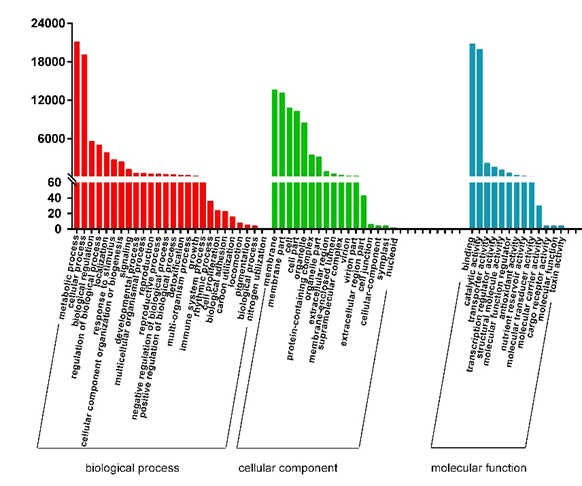


Fig.S2


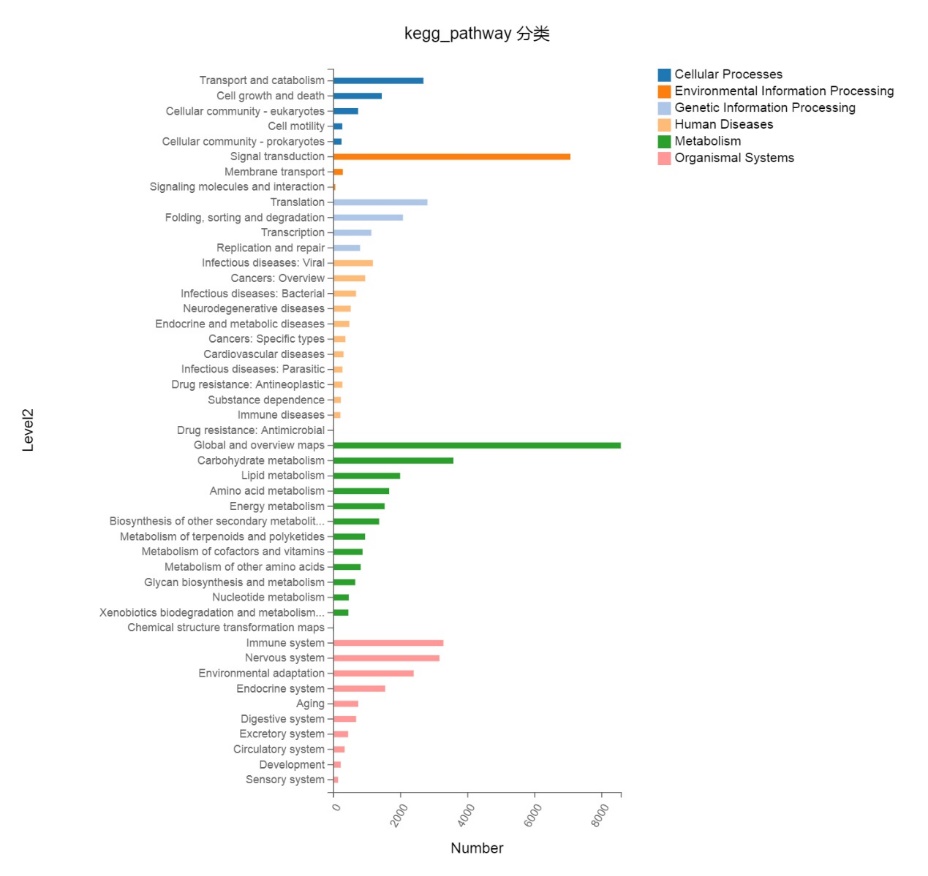

Supplement: Supplementary file 1 — Additional file 1: Fig. S1. GO classification of unigenes. In this figure, the abscissa represents the secondary classification of GO terms; the ordinate represents the number of genes associated with the secondary classification; and the three colors represent three classifications, including biological processes (red), cellular components (green), and molecular function (blue). Fig. S2. The KEGG annotation of unigenes. Permissions to use the KEGG pathway map was taken from the Kanehisa Laboratories (https://www.kanehisa.jp/). [file 12870_2022_4010_MOESM1_ESM.docx]
